# Optimizing therapeutic outcomes: preconditioning strategies for MSC-derived extracellular vesicles

**DOI:** 10.3389/fphar.2025.1509418

**Published:** 2025-02-10

**Authors:** Yuqi Song, Fengrui Liang, Weikun Tian, Erin Rayhill, Liping Ye, Xinghan Tian

**Affiliations:** ^1^ School of Clinical Medicine, Shandong Second Medical University, Weifang, Shandong, China; ^2^ Biology Department, Hamilton College, Clinton, NY, United States; ^3^ Yantai Yuhuangding Hospital, Yantai, Shandong, China

**Keywords:** miRNA, MSC-EVs, exosomes, preconditioning strategies, MSCs (mesenchymal stem cells)

## Abstract

Mesenchymal stem cells (MSCs) and MSC-derived extracellular vesicles (MSC-EVs) are increasingly recognized for their therapeutic potential in regenerative medicine, driven by their capabilities in immunomodulation and tissue repair. However, MSCs present risks such as immunogenic responses, malignant transformation, and the potential to transmit infectious pathogens due to their intrinsic proliferative and differentiative abilities. In contrast, MSC-EVs, particularly exosomes (MSC-exosomes, 30–150 nm in diameter), offer a safer therapeutic profile. These acellular vesicles mitigate risks associated with immune rejection and tumorigenesis and are inherently incapable of forming ectopic tissues, thereby enhancing their clinical safety and applicability. This review highlights the therapeutic promise of MSC-exosomes especially focusing on the modulation of miRNA (one of bioactive molecules in MSC-EVs) profiles through various preconditioning strategies such as exposure to hypoxia, chemotherapeutic agents, inflammatory cytokines, and physical stimuli. Such conditioning is shown to optimize their therapeutic potential. Key miRNAs including miR-21, miR-146, miR-125a, miR-126, and miR-181a are particularly noted for their roles in facilitating tissue repair and modulating inflammatory responses. These functionalities position MSC-exosomes as a valuable tool in personalized medicine, particularly in the case of exosome-based interventions. Despite the potential of MSC-EVs, this review also acknowledged the limitations of traditional MSC therapies and advocates for a strategic pivot towards exosome-based modalities to enhance therapeutic outcomes. By discussing recent advances in detail and identifying remaining pitfalls, this review aims to guide future directions in improving the efficacy of MSC-exosome-based therapeutics. Additionally, miRNA variability in MSC-EVs presents challenges due to the diverse roles of miRNAs play in regulating gene expression and cell behavior. The miRNA content of MSC-EVs can be influenced by preconditioning strategies and differences in isolation and purification methods, which may alter the expression profiles of specific miRNAs, contributing to differences in their therapeutic effects.

## 1 Introduction

Mesenchymal stem/stromal cells (MSCs) are non-hematopoietic multipotent stem cells known for their self-renewal capacity and ability to differentiate into various lineages. These cells are integral to tissue regeneration and maintaining homeostasis, while also exhibiting significant immunomodulatory properties. As such, MSCs are pivotal in therapeutic strategies aimed at enhancing tissue repair and modulating immune responses (Wang et al., 2014). However, recent studies have revealed that the application of MSCs in tissue support and immune regulation is constrained by their low engraftment rates and short lifespan (Moll et al., 2019). Consequently, there is growing interest in the therapeutic potential of extracellular vesicles released by MSCs (MSC-EVs). MSC-EVs enhance therapeutic outcomes for a range of diseases and mitigate the risk of immune rejection. In particular, there is evidence that MSC-EVs ability to modulate various immune cells, providing therapeutic benefits in inflammatory diseases (Liu et al., 2023). Their therapeutic potential spans a broad spectrum of conditions, highlighting the importance of further investigation into their mechanisms of action and optimal applications (Martí‐Chillón et al., 2023; Adamiak et al., 2018).

EVs are pivotal in intercellular communication, carrying a range of bioactive molecules including proteins, lipids, and nucleic acids [microRNAs (miRNAs) and mRNA], crucial in mediating the therapeutic effects traditionally attributed to parent MSCs (Lee and Kim, 2022; Zhu et al., 2023a). Exosomes, distinct from other EVs such as microvesicles and apoptotic bodies, excel in therapeutic applications due to their unique biogenetic origin and nano-scale size (30–150 nm) (Rezaie et al., 2022). Arising from endosomal compartments, exosomes exhibit specific molecular profiles that facilitate targeted interaction and uptake by recipient cells. This selective cargo loading and inherent stability in circulation position exosomes as superior candidates for precision drug delivery and regenerative therapies. They offer enhanced delivery efficiency and reduced immunogenicity compared to larger EVs, whose formation and content are less controlled. The high specificity and customizable nature of exosomes make them highly valuable in advancing personalized medicine strategies (Gurunathan et al., 2019). Exosomes derived from MSCs (MSC-exosomes) are increasingly recognized as potential cell-free therapies for various diseases, notably due to the miRNAs encapsulated in these EVs because they play pivotal roles in various biological processes (Wang et al., 2018). Ongoing studies continue to demonstrate that the miRNA profiles of MSC-exosomes are not static but rather dynamic, significantly influenced by external processing conditions. This variability underscores the complexity of exosome-based therapeutics, highlighting the need for precise characterization and modulation of their miRNA content to enhance therapeutic efficacy (Kurian et al., 2021).

miRNAs are non-coding RNAs approximately 21–23 nucleotides in length that regulate gene expression by binding to the 3′ untranslated region (UTR) of target mRNAs (Rossi, 2011). MSC-EVs are increasingly recognized for their stability and safety compared to parent MSCs, particularly in clinical applications. While MSC-EVs hold promise in regenerative medicine, variability in miRNA profiles poses challenges for therapeutic consistency. Addressing this, preconditioning strategies play a pivotal role in modulating miRNA content to align with therapeutic goals. With advancements in high-throughput gene sequencing technology, differences in miRNA expression profiles in MSC-exosomes can be identified under various stimulatory conditions (Leidal and Debnath, 2020). This provides powerful tools for a comprehensive understanding of the regulatory mechanisms of miRNAs under different processing conditions. For instance, conditions such as hypoxia, stimulation by inflammatory factors, and chemical or physical stimuli have been reported to significantly alter the miRNA expression profiles in exosomes (Strecanska et al., 2024). Alterations in miRNA expression profiles in exosomes can significantly impact their biological functions and consequent therapeutic outcomes. This review seeks to collate and critically evaluate the literature concerning variations in miRNA content in exosomes secreted by MSCs across diverse treatment conditions. We will investigate the correlations between these miRNA alterations and exosomal functions as well as discuss potential therapeutic leveraging miRNA regulatory mechanisms. Our analysis aims to optimize the use of MSC-exosome therapeutics in various disease.

Herein, we explore the connection between these miRNA changes in MSC-exosomes and exosome functions, and discuss their regulatory mechanisms during treatment with MSC-exosomes in order to optimize the manufacture and generation of MSC-exosomes in the future. This review emphasizes the regulatory and clinical landscape for MSC-EVs, focusing on their role in inflammatory and immune diseases and the importance of quality control in therapeutic applications.

## 2 Pre-conditioning strategies for MSCs

Conditioning MSCs to enhance the therapeutic potential of their EVs through miRNA modulation involves a variety of strategies. These approaches aim to manipulate the cellular environment or apply physical stimuli to induce specific changes in the miRNA profile of the EVs, thereby optimizing their regenerative and immunomodulatory functions.

### 2.1 Biological modulators

#### 2.1.1 Lipopolysaccharide

LPS is a potent endotoxin derived from the outer membrane of Gram-negative bacteria and is commonly used in research to simulate inflammatory conditions. LPS is commonly used in MSC conditioning due to its potent ability to activate the immune response. Notably, the application of LPS, at low doses, exhibits protective effects against numerous diseases. Different doses of LPS induce MSCs to secrete exosomes with distinct mechanisms of action, likely due to varying miRNA content at each dose level. For instance, Zhang, P. et al. observed that stimulating BMSCs with 0.1 μg/mL LPS enhanced the expression of miR-222-3p in exosomes (Zhang et al., 2023). Similarly, Liu, H.-Y. et al. found that a dose of 0.5 μg/mL LPS increased the expression of miR-181a-5p in BMSC exosomes (Liu et al., 2020a). Furthermore, Zheng, T. et al. applied 1 μg/mL LPS to BMSCs, altering the miRNA expression profile in EVs and upregulating miR-150-5p (Zheng et al., 2024). These findings illustrate that varying LPS concentrations lead to different miRNA profiles in exosomes and distinct biological effects, yet all contribute to mitigating inflammatory damage. Low doses of LPS induce MSCs to secrete exosomes with distinct miRNA profiles and biological effects, highlighting dose-dependent responses that mitigate inflammatory damage. Given that low doses of LPS have inherent protective effects in disease treatment, it prompts the investigation into whether other compounds might also exhibit dose-dependent differential responses in MSC-derived exosomes.

#### 2.1.2 Inflammatory cytokines and growth factors

The primary function of immune checkpoints (ICPs) is to prevent the initiation of adverse reactions and to regulate immune responses, thereby maintaining homeostasis. ICPs are produced by various types of immune regulatory cells, and deficiencies in their expression or function can result in overactive immune responses, potentially leading to autoimmune diseases. MSCs contribute to immune regulation by producing ICPs, immune checkpoint ligands (ICPLs), and modulating immune cell responses via secretion and direct interactions (Hazrati et al., 2024). Pretreatment of MSCs in inflammatory conditions enhances their therapeutic potential. promoting anti-inflammatory cytokine production and increasing ICPL expression. MSC-derived EVs encapsulate miRNAs that significantly influence immune modulation, offering strategies for regenerative medicine and inflammatory disease treatment.

##### 2.1.2.1 TNF-α

TNF-α initiates the inflammatory response and collaborates with various factors to participate in inflammation and tissue repair. TNF-α significantly influences the fate and functional reprogramming of MSCs in the inflammatory microenvironment, thereby enhancing their immune regulatory and tissue repair capabilities (Li W. et al., 2023). Although some studies have found that the protein content of exosomes increased after TNF-α stimulation of MCSs, which can directly promote the polarization of macrophages, it is undeniable that miRNAs also play a crucial role in this process (Harting et al., 2018). Liang, Y.-C. et al. conducted a study and found that low-dose TNF-α (10 ng/mL) stimulation of human umbilical cord mesenchymal stem cells (hucMSCs) led to an increase in the content of miR-146a in exosomes (Liang et al., 2019). In another study using higher dose of TNF- α, Domenis, R. et al. found that human adipose-derived mesenchymal stem cells (hadMSCs) stimulated by TNF-α (20 ng/mL) not only showed an increased content of miR-146a in exosomes but also a notable rise in miR-34 levels (Domenis et al., 2018). These findings suggest a dose-dependent response, with higher TNF-α concentrations amplifying miRNA alterations in exosomes and enhancing immunomodulatory effects.

Moreover, IL-1β stimulation of bone marrow MSCs (BMSCs) increased miR-146a in EVs, promoting macrophage polarization and improving organ injury in sepsis (Song et al., 2017). Gingival tissue-derived MSCs (GMSCs) showed increased miR-21-5p under low-dose TNF-α (10 ng/mL) (Yu et al., 2022) and miR-1260b under high-dose (100 ng/mL) stimulation (Nakao et al., 2021). However, moderate doses of TNF-α may inhibit cell proliferation and promote autophagy and apoptosis, raising questions about the practical applicability of high-dose stimulation (Li W. et al., 2023). Additionally, miR-299-3p and miR-24-3p were upregulated in hucMSC (Zhang et al., 2020) and menstrual blood MSC (MenSC) exosomes under 20 ng/mL TNF-α stimulation (Xu et al., 2023).

These studies underscore the specificity of miRNA responses to TNF-α across MSC sources, suggesting tailored strategies for therapeutic applications. Future research should explore the dose-dependent effects and verify findings in human MSCs for clinical use.

##### 2.1.2.2 IFN-γ

IFN-γ alone or combined with TNF-α enhances MSC immunosuppressive capacity (Harting et al., 2018; Chen et al., 2024). Moreover, the application of 50 ng/mL IFN-γ to stimulate BMSCs increases the levels of miR-125a and miR-125b in their exosomes (Yang et al., 2020). Ragni et al. performed sequencing analysis on adipose-derived stem cells (ASCs) stimulated with IFN-γ (10 ng/mL). The study identified that secreted molecules and miRNAs can promote M2 macrophage polarization and reduce the inflammation marker VCAM-1 in chondrocytes (Ragni et al., 2020). The overexpressed miRNAs included miR-146b-5p, miR-146b-3p, miR-155-5p, miR-210-3p, miR-29b-3p, miR-455-5p, and miR-886-3p. IFN-γ was found to decrease the expression of miR-149, which is involved in inflammation, while increasing miR-210 levels.

##### 2.1.2.3 IL-6 and MIF

Although most studies on IL-6 focus on its secretion in MSC-derived EVs, stimulation with IL-6 (1 ng/mL) increased miR-455-3p expression in hucMSC-derived exosomes (Shao et al., 2020). Meanwhile, macrophage migration inhibitory factor (MIF) has emerged as a novel stimulus. MIF-stimulated MSCs showed upregulation of LncRNA-NEAT1 in exosomes, highlighting its therapeutic potential (Zhuang et al., 2020).

These findings highlight the complexity of miRNA responses to inflammatory cytokines, emphasizing the need for standardized protocols in MSC pretreatment to optimize therapeutic efficacy. The heterogeneity of miRNA profiles necessitates careful selection of cytokine dosage and MSC sources. Dose-dependent and synergistic effects should be further investigated to refine therapeutic applications.

In summary, miRNAs in MSC-derived EVs are central to immune regulation and regenerative medicine. Future research should prioritize exploring combined stimuli, verifying dose-dependent effects, and identifying optimal conditions for clinical applications.

#### 2.1.3 Oxidative and sulfide compounds

Oxidative and sulfide compounds play a critical role in modulating the biological activity of MSCs and their secreted EVs. These compounds influence the expression and packaging of miRNAs within EVs, resulting in altered miRNA profiles that enhance their antioxidative, anti-inflammatory, and cytoprotective properties.

Hydrogen peroxide (H₂O₂) is utilized to stimulate MSCs to modulate miRNA profiles. Specifically, treatment of bone marrow-derived MSCs (BMSCs) with 100 μM H₂O₂ elevates the levels of miR-21 in the EVs they secrete, which aids in reducing H₂O₂-induced apoptosis (Shi et al., 2018). Additionally, hydrogen sulfide (H₂S), known for its protective effects on central nervous system injuries, enhances the neuroprotective and anti-inflammatory functions of microglia and monocyte macrophages by upregulating miR-7b-5p in MSC-derived EVs when used as a pre-treatment (Chu et al., 2020). Nitric oxide (NO) has been shown to increase levels of vascular endothelial growth factor (VEGF) and miR-126 in exosomes derived from human placenta-derived MSCs, promoting angiogenesis (Du et al., 2017). MiR-126 is responsive to various stimuli, including hypoxia.

Heme oxygenase-1 (HO-1), a stress-inducible protein abundantly expressed in tissues, catalyzes the breakdown of heme into biliverdin, free divalent iron, and carbon monoxide (CO). This enzymatic activity prevents free heme from sensitizing cells to apoptosis, thereby mitigating the development of various immune-mediated inflammatory diseases (Gozzelino et al., 2010). Stimulation of bone marrow-derived mesenchymal stem cells (BMSCs) with 10 μM HO-1 in complete culture medium enhances the miR-183-5p content in secreted exosomes, which subsequently inhibits cardiomyocyte senescence via the HMGB1/ERK pathway (Zheng et al., 2021). Heme oxygenase (HO), the rate-limiting enzyme in heme catabolism, produces equimolar amounts of CO. Direct exposure of human umbilical cord mesenchymal stem cells (hucMSCs) to 250 ppm CO gas for 4 h elevates the levels of miR-145-3p and miR-193a in the EVs, indicating differential miRNA regulation compared to HO-1 stimulation (Hwang et al., 2024). This suggests that while HO-1 facilitates the production of multiple byproducts, its effects differ significantly from direct CO exposure, highlighting the importance of enzyme-specific pathways in cellular responses.

Peroxiredoxin II (Prx II) is an antioxidant enzyme that rapidly quenches low concentrations of intracellular reactive oxygen species (ROS) by stabilizing the mitochondrial membrane potential (Jin et al., 2019). PrxII regulates mesenchymal cell growth through the Wnt/β-catenin signaling pathway (Han et al., 2020). Stimulation of DMSCs (dermal mesenchymal stem cells) leads to downregulation of miR-221 and upregulation of miR-21-5p in the secreted exosomes, which subsequently promotes skin wound healing (Jin et al., 2021).

These findings underscore the importance of selecting appropriate stimuli to tailor miRNA content in MSC-derived EVs, thereby enhancing their therapeutic potential for treating a wide range of conditions from tissue degeneration to inflammatory diseases. Future studies should focus on further elucidating the mechanisms by which these treatments modulate miRNA profiles and their subsequent biological effects.

#### 2.1.4 Pharmacological agents

Various pharmacological compounds and biochemical stimuli can modulate the miRNA content in MSC-(EVs, enhancing their therapeutic potential for a wide range of medical conditions.

Advanced glycation end products (AGEs), which form through non-enzymatic reactions between proteins and glucose, induce vascular complications in diabetes by increasing the secretion of miR-146a in exosomes from BMSCs (Wang et al., 2018; Bodiga et al., 2014). This miRNA is implicated in several chronic diseases, with its dysregulation linked to abnormal levels of pro-inflammatory cytokines (Shahriar et al., 2020). Exposure of BMSCs to the traditional Chinese medicine compound Tongxinluo (TXL) also increases miR-146a-5p levels in exosomes, initially thought to mimic statin effects (Xiong et al., 2022). Similar stimulatory effects are observed with atorvastatin (ATV), which enhances miR-221-3p secretion from human BMSCs (Yu et al., 2020). The impact of LncRNA H19 upregulation by ATV mirrors that seen with AGEs, although targeting different miRNAs (Huang et al., 2019). Furthermore, cardiovascular drugs like nicorandil induce the overexpression of multiple miRNAs such as miR-148a-3p, miR-125a-5p, miR-100-5p, among others, in murine BMSC-derived exosomes, underscoring the diverse miRNA-mediated mechanisms activated by pharmacological stimulation of MSCs (Gong et al., 2024).

Treatment with Buyang Huanwu Decoction (BYHWD), a traditional Chinese medicine, elevates miR-126 levels but also decreases miR-221 and miR-222 expressions (Yang et al., 2015a). Furthermore, incubation of BMSCs with 2.5 mM lithium chloride for 24 h markedly boosts the concentrations of miR-132 and miR-1906 in the EVs (Haupt et al., 2020). MiR-132 has been associated with cardioprotective effects in myocardial infarction (Ma et al., 2018), while miR-1906 offers neuroprotection in stroke treatment, highlighting the diverse therapeutic potential of modulating miRNA profiles in MSC-derived EVs across various medical conditions.

Tropoelastin (TE) has been observed to reduce wound healing duration and exhibit anti-inflammatory properties (Wang et al., 2024a). In osteoarthritis treatment, exosomes derived from MSCs stimulated with tropoelastin demonstrate enhanced therapeutic effects compared to the direct application of TE. This superior efficacy is attributed to the increased expression of miR-451-5p within the exosomes (Meng et al., 2023). Melatonin treatment of MSC resulted in increased miR-18a-5p content in EV and reduced hyperoxy-induced lung injury (Zou et al., 2024).

Certain biomolecules possess intrinsic therapeutic properties for various diseases, which are further amplified when MSCs are stimulated. This amplification is closely associated with alterations in the miRNA content of the cells. These changes suggest that exosomes derived under such stimulatory conditions hold potential for broader applications in treating additional diseases. Although further validation is necessary due to the limited number of studies, the current evidence strongly supports ongoing research to unlock the full therapeutic potential of this approach.

### 2.2 Hypoxia

The environment in which MSCs are cultured can significantly affect their proliferation, differentiation, and therapeutic potential. Unlike conventional cultured cells *in vitro*, MSCs are typically exposed to hypoxic conditions *in vivo*. Thus, studying MSCs under hypoxia can enhance the understanding of exosome secretion from these cells (Mohyeldin et al., 2010). An oxygen concentration range of 1%–5% is usually used to simulate an oxygen-deficient environment. However, even within this range, variations in oxygen concentration can lead to different effects on regulating miRNA expression under hypoxic conditions. Hypoxia profoundly influences the exosomes secreted by MSCs upon stimulation, with its impacts being extensively studied in the field of regenerative medicine.

#### 2.2.1 Human MSCs

Experimental studies have demonstrated that hypoxia preconditioning plays a crucial role in tissue damage repair. In a study investigating exosomal miR-126 from human umbilical cord mesenchymal stem cells (hucMSCs) (Liu et al., 2020b), it was found that HIF-1α activation in hucMSCs under 1% oxygen conditions resulted in an increased miR-126 concentration in exosomes, enhancing endothelial cell proliferation, angiogenesis, and migration. Similarly, under the same hypoxic conditions (1% O₂ at 37°C for 48 h), levels of miR-17-5p (Chu et al., 2023) and miR-7-5p (Hu et al., 2023) in hucMSC-derived exosomes were significantly elevated. These findings highlight the adaptive roles of hucMSCs in modifying miRNA profiles in exosomes under specific environmental conditions, thereby driving targeted cellular responses essential for tissue regeneration.

Several studies have documented that members of the let-7 miRNA family are upregulated in human adipose-derived mesenchymal stem cells (hADSCs) under hypoxic conditions (5% O2) (Zhu et al., 2020). However, alterations in this hypoxic environment led to the downregulation of specific let-7 miRNAs, including LET-7i-5P, LET-7A-5P, LET-7F-5P, alongside miR-125a-5p and miR-26a-5p (Koch et al., 2022). Despite the consistent overexpression of the let-7 family under hypoxia, its relevance to certain disease treatments has limited its focus in further investigations. Notably, the overexpression of let-7 under such conditions remains significant. Studies on MSCs predominantly centers on bone marrow-derived MSCs, with a relatively smaller focus on human-derived MSCs, which typically necessitate confidentiality regarding donor information. Additionally, under hypoxic conditions, miR-424 levels are found to increase in EVs, a finding supported by experimental evidence (Mathew et al., 2023). Under 5% oxygen, although exosomal miRNAs such as miR-181c-5p, miR-18a-3p, miR-376a-5p, and miR-337-5p are downregulated, they continue to exhibit therapeutic potential (Zhang B. et al., 2022).

#### 2.2.2 Mouse MSCs

In addition to human mesenchymal stem cells (MSCs), mouse MSC are frequently utilized due to their accessibility and utility in disease modeling for experimental studies, facilitating the validation of EV therapeutics. Under 1% oxygen conditions, miR-126a-5p expression is upregulated in BMSC-derived exosomes, influencing microglial polarization (Liu et al., 2020c) and chondrocyte proliferation (Rong et al., 2021). Similarly, miR-17-5p in BMSC-derived exosomes is involved in regulating nucleus pulposus cells (Zhou et al., 2022), and miR-205-5p enhances cartilage repair when used with injectable silk fibroin hydrogels (SF/ACs/H-Exos) (Shen et al., 2022). Under 3% O₂ conditions, miRNA-421-3p expression in EVs was increased (Deng et al., 2023), alongside elevated levels of circRNA_Nkd2 (Wang et al., 2024b) and lncRNA XIS T (Ren et al., 2023) in EVs under hypoxic conditions. Mao, C.-Y. et al. reported that miR-224-5p levels in exosomes from hypoxically cultured mouse ADSCs were increased, which alleviated early myocardial ischemia (Mao et al., 2022). The study also found that miR-21 levels were elevated at 1% O₂ (Li et al., 2024). These studies, utilizing high-throughput sequencing, revealed that miRNA expression is not limited to singular upregulation but encompasses a broad range of differential gene expression, and the remaining unstated miRNAs can be search in the table (Table 1). Hypoxic conditions also elevate the expression of circRNAs such as circ-Scmh1 and circ-Erbb2ip, suggesting a significant regulatory role of circRNAs in therapeutic applications. Reviewing miRNA expression changes in exosomes from hypoxia-preconditioned MSCs underscores the potential for expansive study across various diseases. For example, hypoxia plays a significant role in the tumor microenvironment, raising questions about whether hypoxia could contribute to cancer therapy (Jahangiri et al., 2023).

**TABLE 1:** Effects of different pre-conditioning approaches on miRNA profiles, miRNA targets and functions.

Pretreatment	miRNA	Target & pathway	Function	MSC	Reference
1% O_2_	miR-126	SPRED1/Ras/Erk	Promotes angiogenic fracture healing	hucMSC	Liu et al. (2020b)
1% O_2_	miR-17-5p	TLR4/ROS/MAPK	Blocking NET formation	hucMSC	Chu et al. (2023)
1% O_2_	miR-423-5p↑, miR-7-5p↑, miR-708-5p↑, miR-483-5p↓, miR-891a-5p↓, miR-652-5p↓	p65/TNF-α/NF-κB/Cxcl2	Inflammatory microenvironment involved in disc degradation promotes proliferation of nucleus pulposus cells, enhances proteoglycan synthesis and collagen formation	hucMSC	Hu et al. (2023)
1% O_2_ & Reduce the nutrient content of the medium (e.g., glucose, amino acids, etc.)	miR-181c-5p↑, miR-18a-3p↑, miR-376a-5p↑, miR-337-5p↑	—	Promotes angiogenesis and tissue regeneration	hucMSC	Zhang B. et al. (2022)
Serum-and glucose-deprived	miR-29a-3p	CTNNBIP1/Wnt/β-catenin	Promote angiogenesis	hucMSC	Wu et al. (2024b)
5% O_2_	let-7b-5p↑, let-7f-5p↑, let-7a-5p↑, let-7i-5p↑, let-7c-5p↑, let-7e-5p↑, let-7g-5p↑, let-7d-5p↑	let-7/AGO1/VEGF	Promote the survival of fat grafts; activity of proliferation, migration and tube formation in HUVECs	hADSC	Zhu et al. (2020)
1% O_2_	let-7i-5P↓, let-7A-5P↓, let-7F-5P↓, let-7F-5P↓, miR-125a-5p↓, miR-26a-5p↓	—	Anti-inflammatory effect on epithelial cells	hADSC	Koch et al. (2022)
1% O_2_	miR-424↑, (miR-7i↑, miR-455↑, miR-19a↑, miR-19b↑, miR-146b↑, miR-27b↑, miR-210↑, miR-380) ↑	—	Reducing inflammatory cytokine production in retinal microglia, and attenuating oxygen free radicals in Muller cells and microvascular endothelial cells	hBMSC	Mathew et al. (2023)
1% O_2_ & XFS	miR-214↑, miR-145↑ (miR-21↑, let-7b-5p↑, miR-301a-3p↑, miR-30b-5p↑, miR-30c-5p↑)	Wnt, TGF-beta and PI3K-Akt (DIANA myRPath 3.0.)	IL-1α-induced inflammation and to reduce production of pro-inflammatory cytokines	hBMSC	Palamà et al. (2023)
5% O_2_	miR-181c-5p↓, miR-18a-3p↓, miR-376a-5p↓, miR-337-5p↓	miRNA-18-3P/JAK-STAT; miRNA-181c-5p/MAPK	Promote proliferation and migration and inhibit apoptosis	hBMSC	Zhang B. et al. (2022)
Serum-free	miR-17-92↑	—	Accelerated cell proliferation, migration, angiogenesis, and enhanced against erastin-induced ferroptosis *in vitro*	hBMSC	Nie et al. (2023)
1% O_2_ & 10% Mill Creek Life Sciences	miR-23a↑, miR-125b↑, miR-199a↑, miR-199b↑, miR-4454↑, miR-7975↑	—	Endocytosis, immune response, inflammation, osteogenesis, osteoblast differentiation, and cell proliferation	hBMSC, hucMSC, hHDCs	Vaka et al. (2023)
1% O_2_	miR-216a-5p↑ (miR-99b-5p, miR-301a, miR-126, miR-210-3p)	TLR4/NF-κB/PI3K/AKT	Microglia M1/M2 polarization	BMSC	Liu et al. (2020c)
1% O_2_	miR-216a-5p↑	JAK2/STAT3	Promote the proliferation, migration, and apoptosis inhibition of chondrocytes	BMSC	Rong et al. (2021)
1% O_2_	miR-17-5p	TLR4/PI3K/AKT	Modulate proliferation and synthesis of nucleus pulposus cells (NPCs) matrix	BMSC	Zhou et al. (2022)
1% O_2_	miR-205-5p	PTEN/AKT	Promote cartilage regeneration	BMSC	Shen et al. (2022)
3% O_2_	miRNA-421-3p	mTOR/ULK1/FUNDC1	Activating autophagy	BMSC	Deng et al. (2023)
0% O_2_	miR-224-5p	TXNIP/HIF-1α	Improve myocardial hypoxia tolerance	ADSC	Mao et al. (2022)
1%O_2_	miR-21-5p (miR-1-3p)	SPRY1/PI3K/AKT	HUVEC proliferation, migration, and angiogenesis	ADSC	Li et al. (2024)
TNF-α (10 ng/mL)	miR-146a↑	—	Reduced antifibrotic effects	hucMSC	Liang et al. (2019)
TNF-α (10 ng/mL)	miR-21-5p↑	PDCD4	Alleviating apoptosis	GMSC	Yu et al. (2022)
TNF-α (20 ng/mL)	miR-34↑, miR-146a↑	Notch1/IRAK1	Enhance M2 macrophage polarization	hADSC	Domenis et al. (2018)
TNF-α (100 ng/mL)	miR-1260b↑	Wnt5a/RANKL	Enhance M2 macrophage polarization and inhibit periodontal bone loss	GMSC	Nakao et al. (2021)
TNF-α (20 ng/mL)	miR-299-3p↑	NLRP3	Attenuates inflammatory damage caused and promotes liver tissue repair	hucMSC	Zhang et al. (2020)
TNF-α (20 ng/mL)	miR-24-3p↑	Interferon regulatory factor 1 (IRF1)	Promoted the polarization of M2 macrophages	MenSC	Xu et al. (2023)
IL-1β (10 ng/mL)	miR-146a↑	—	Enhance M2 macrophage polarization	BMSC	Song et al. (2017)
IFN-γ (50 ng/mL)	miR-125a↑, miR-125b↑	Stat3	Repress Th17 cell differentiation	BMSC	Yang et al. (2020)
IFN-γ (10 ng/mL)	miR-146b-5p, miR-146b-3p, miR-155-5p, miR-210-3p, miR-29b-3p, miR-455-5p, and miR-886-3p↑	—	Modulated the polarization and inflammatory response of macrophage	ADSC	Ragni et al. (2020)
IL-6 (1 ng/mL)	miR-455-3p↑	PIK3r1	Suppress monocyte/macrophage activation and alleviate acute liver injury	hucMSC s	Shao et al. (2020)
rhGDF7 (50 ng/mL)	miR-369-3p↑	PDE4D/AMPK	Preventing I/R-induced inflammation, oxidative stress and neural damage	BMSC	Wang et al. (2022)
Tropoelastin	miR-451-5p↑	—	Maintain Chondrocyte Phenotype, Formation of Cartilage Extracellular Matrix	ADSC	Meng et al. (2023)
Peroxiredoxin II	miR-221↓, miR-21-5p↑	—	Wound healing	DMSCs	Jin et al. (2021)
LPS (0.1ug/mL)	miR-222-3p↑	NF-κB/NLRP3/procaspase-1/IL-1β	The polarization of M1 macrophages while increasing the proportion of M2 cells	BMSC	Zhang et al. (2023)
LPS (0.1ug/mL)	miR-181a-5p↑	Irs1/PI3K/Akt/mTOR	Modulated the polarization and inflammatory response of macrophage	BMSC	Liu et al. (2020a)
LPS (0.5ug/mL)	miR-150-5p↑	ATF2	Inhibits myocardial inflammation and oxidative stress	BMSC	Zheng et al. (2024)
H₂O₂ (100 µM)	miR-21↑	PTEN/PI3K/AKT	Protection against oxidative stress-triggered cell death	BMSC	Shi et al. (2018)
H₂S (1 μM)	miR-7b-5p↑	FOS	CD45 low microglia and CD45 high brain mononuclear phagocytes toward a beneficial phenotype	BMSC	Chu et al. (2020)
NO	miR-126↑			hP-MSCs	Du et al. (2017)
CO (250ppm)	miR-145-3p↑, miR-193a-3p↑	—	Enhance autophagy	huc-MSC	Hwang et al. (2024)
Hemin (10 μM)	miR-183-5p↑	HMGB1/ERK pathway	Enhance the cardioprotective effects by regulating mitochondrial fission	hBMSC	Zheng et al. (2021)
AGEs (200 μg/mL)	miR-146a↑	TXNIP	Increased ROS production in VSMCs promotes their osteogenic differentiation	BMSC	Wang et al. (2018)
Tongxinluo (TXL) (400 μg/mL)	miR-146a-5p↑	IRAK1/NF-κB p65 pathway	Protect H9C2 cells against hypoxic injury	BMSC	Xiong et al. (2022)
Atorvastatin (1 μM)	miR-221-3p↓	AKT/eNOS pathway	Proliferation, migration, tube formation, and VEGF secretion in endothelial cells	hBMSC	Yu et al. (2020)
Nicorandil (200 μmol/L)	miR-125a-5p↑	TRAF6/IRF5 signaling pathway	Cardiac repair effects and macrophage polarization toward M2 phenotype	BMSC	Gong et al. (2024)
Low-intensity pulsed ultrasound (90 mW/cm^2^)	miR-935↑	—	Osteogenic differentiation and anti-inflammation	SCAP	Zhang T. et al. (2022)
Low-intensity pulsed ultrasound (300 mW/cm^2^)	miR-487b-3p↑, miR-328-5p↑	MAPK pathway	Anti-inflammatory phenotype	BMSC	Li W. et al. (2023)
50 μg/mL Fe_3_O_4_ and a 100 mT SMF	miR-1260a↑	HDAC7 and COL4A2	Bone regeneration and angiogenesis	hBMSC	Wu et al. (2021)
Blue (455 nm) monochromatic light	miR-135b-5p↑, miR-499a-3p↑	MEF2C signaling	HUVEC migration and vessel formation	hucMSC s	Yang et al. (2019)

Hypoxia significantly alters the secretion and miRNA content of exosomes from MSCs, enhancing their therapeutic potential, particularly in regenerative medicine. Studies have demonstrated that hypoxic conditions (1%–5% O₂) modulate the miRNA profiles within MSC-derived exosomes, with specific miRNAs like miR-126 and miR-17-5p showing increased expression.

Although hypoxia remains the primary focus in MSC preconditioning, the influence of other environmental conditions, such as pH and thermal stress, should not be overlooked. Limited research exists on MSC-EVs under these conditions, but one study has shown that thermal stress can significantly enhance the adhesive potential, migratory capacity, surface marker expression, and multilineage differentiation of MSCs, albeit with reduced proliferation. Notably, this preconditioning was found to boost MSCs’ tumor-targeting capabilities (Rühle et al., 2020). Given the sparse data on the impact of thermal stress and other environmental factors on MSC-EVs, additional studies are required to determine the potential research prominence of these factors.

### 2.3 Physical stimuli

Various physical modalities, including ultrasound, electrical stimulation, and ionizing radiation, have been shown to augment the secretion of MSC-derived exosomes or mimetic nanoparticles. This enhancement facilitates large-scale production and modifies biological functionality through differential miRNA expression (Wu et al., 2024a). Specifically, low-intensity pulsed ultrasound (LIPUS), a non-invasive mechanical stimulus with a power density significantly lower than conventional ultrasound, at an intensity of 90 mW/cm^2^, can increase miR-935 content in stem cells from the apical papilla (SCAP) EVs following a 30-min exposure (Zhang T. et al., 2022). When LIPUS is applied to BMSCs at 300 mW/cm^2^, there is a 3.66-fold increase in the release of EVs, with enhanced IL-10 levels and elevated expressions of miR-328-5p and miR-487b-3p (Li X. et al., 2023). Additionally, the presence of 50 μg/mL Fe_3_O_4_ nanoparticles combined with a 100 mT static magnetic field (SMF) markedly increases exosome production and upregulates miR-143-3p, miR-23a-3p, miR-1260a, and miR-3960 (Wu et al., 2021). In terms of photostimulation, blue light (455 nm) is more efficacious than red light (638 nm) in promoting wound healing and upregulating miR-135b and miR-499a in exosomes (Yang et al., 2019). Moreover, other physical stimuli such as mechanical forces, ionizing radiation, and electrical pulses remain underexplored and may offer additional avenues for enhancing the yield and functionality of MSC-derived EVs. Investigating and harnessing these physical factors could optimize the clinical application and efficacy of MSC-EVs, potentially leading to improved therapeutic outcomes.

### 2.4 Nutritional and metabolic stress

Incorporating or removing different nutrients from the culture medium can significantly impact the characteristics of EVs produced by MSCs, making it an important research direction. Fitzgerald, J. C.et al. highlighted significant differences in clonal formation, proliferation, differentiation potential, and immunomodulatory capacity of MSCs depending on the culture media used. Specifically, for hBMSCs, the protein profiles of EVs varied markedly under different media conditions (Fitzgerald et al., 2023), although miRNA content was not extensively examined.

#### 2.4.1 Serum-free media

Culturing MSCs under serum-free conditions has emerged as a significant approach due to its impact on various cellular functions and exosome composition (Giannasi et al., 2023). These modifications enhance the therapeutic potential of MSC-EVs, positioning serum-free conditions as a promising strategy for regenerative medicine. Serum-free culture media have been shown to influence mitochondrial antioxidant functions and alter metabolic products in ASCs. In hBMSCs, the application of serum-free media resulted in an increase in EV-associated miR-17-92 (Nie et al., 2023). Similarly, when HUCMSCs were cultured in serum-free media with glucose deprivation, an elevated level of miR-29a-3p was observed in the EVs (Wu et al., 2024b). Furthermore, combining hypoxia with nutrient-reduced culture medium led hucMSC to increased levels of miR-181c-5p, miR-18a-3p, miR-376a-5p, and miR-337-5p in exosomes (Zhang B. et al., 2022), which promoted chondrocyte proliferation and migration. These findings underscore the research significance of serum-free conditions, highlighting their potential to modulate the miRNA content of EVs and enhance their therapeutic efficacy.

#### 2.4.2 Nutrient modification

The addition of various substances to specialized culture media is crucial for enhancing the quantity of EVs secreted by MSCs due to its ability to influence cellular functions, metabolic processes, and the miRNA composition of EVs, and several methods have proven effective in this regard. A common approach is to supplement the culture medium with platelet lysate and growth factors. For instance, when 10% platelet lysate (Mill Creek Life Sciences) is added to the medium under 1% hypoxic conditions for 48 h, different MSC sources, including hBMSCs, heart-derived cells (HDCs), and hUC-MSCs, exhibit overexpression of miRNAs such as miR-23a, miR-125b, miR-199a, miR-199b, miR-4454, and miR-7975. These miRNAs are implicated in processes like endocytosis, immune response, inflammation, osteogenesis, osteoblast differentiation, and cell proliferation (Vaka et al., 2023).

Additionally, incorporating a specialized xeno-free supplement (XFS) into the culture medium induces the overexpression of miR-145 and miR-214 in EVs, further enhancing their regulatory and therapeutic functions (Palamà et al., 2023). The effects observed with XFS supplementation indicate influences beyond hypoxia alone, underscoring the complexity of culture conditions.

Growth Differentiation Factor 7 (GDF7), also known as BMP12 and CDMP3, is a member of the transforming growth factor-β (TGF-β) superfamily, which is vital for various biological processes, including cell differentiation, survival, embryonic development, and tumorigenesis (Wang et al., 2022). GDF7 significantly contributes to cartilage regeneration and injury repair via the TGF-β signaling pathway (Kong et al., 2023). When bone marrow-derived mesenchymal stem cells (BMSCs) are exposed to recombinant human GDF7 (rhGDF7) at a concentration of 50 ng/mL, the resulting exosomes are enriched with miR-369-3p, suggesting their potential therapeutic application in treating cerebral ischemia/reperfusion (I/R) injury (Tao et al., 2023).

In summary, several approaches have been discussed here to facilitate changes in miRNA content in EVs, enhancing their therapeutic potential. Recently, some studies have discovered more preconditioning methods that can selectively alter the expression of specific miRNAs. Engineering MSC-EVs has emerged as a promising acellular therapeutic approach, utilizing techniques such as CRISPR/Cas9, co-transfection, lentiviral and adenoviral transduction, nanoparticle carriers, and electroporation (Christoffers et al., 2024). CRISPR/Cas9 allows for precise genome editing by introducing the Cas9 nuclease and specific guide RNAs, enabling targeted miRNA modulation. Other miRNAs, such as miR-200a-3p (Zhang et al., 2024a) and miRNA-223-3p (Zhao et al., 2020), have been incorporated into MSC-EVs through alternative methods, contributing to their therapeutic efficacy in various diseases.

## 3 Challenges in MSC-EV therapy and potential solutions

The therapeutic potential of SC-EVs is significant, but several challenges need to be addressed to optimize their clinical applications.

### 3.1 Source-related variability of MSCs

One of the major limitations in current studies is the variability introduced by MSCs derived from different sources, such as bone marrow, adipose tissue, or umbilical cord. These sources exhibit inherent biological differences that can significantly affect the therapeutic properties of their EVs (Wu et al., 2018). This variability presents a challenge for standardizing preconditioning methods and ensuring consistent results across studies. In view of practical clinical applications, it is suggested that human MSC should be preferentially selected in subsequent studies to reduce the impact of species differences (Darlington et al., 2011). To address these challenges, standardized protocols must be developed to harmonize differences between MSC sources and facilitate reliable comparisons. Moreover, under identical conditions, MSCs from different sources may lead to differential expression of miRNAs, emphasizing the need for more research into preconditioning methods tailored to specific MSC sources to ensure the therapeutic potential of their EVs is fully optimized.

### 3.2 Purification and miRNA variability in MSC-EVs

Ultracentrifugation remains the most commonly used method for MSC-EV purification, though clinical applications have explored the use of purification reagents (Takakura et al., 2024; My et al., 2018). However, the efficacy of these methods has not been fully validated, and further investigation into their comparative effectiveness is necessary. One promising solution is a novel microbead-based immunocapture method, which integrates subpopulation selection, electroporation-mediated miRNA loading, and post-electroporation purification into a unified workflow. This approach can effectively eliminate vesicles that lack miRNAs, enhancing the therapeutic efficacy of MSC-derived EVs and optimizing their application for various diseases (Torabi et al., 2024).

### 3.3 Gene editing for miRNA modulation in MSCs

Developing advanced gene-editing protocols using tools like CRISPR/Cas9 presents a promising avenue to precisely modulate miRNA expression in MSCs, thereby controlling the miRNA composition of their EVs (Zhu et al., 2023b). By knocking out undesirable miRNAs that may promote adverse effects or introducing beneficial miRNAs with proven therapeutic efficacy, researchers can engineer MSCs to produce EVs with specific, disease-targeted miRNA profiles. This approach shows significant potential for directly inducing the overexpression of specific miRNAs in MSC-derived EVs to achieve therapeutic effects. However, its practical feasibility and applicability remain uncertain, as further research and validation are required to confirm its safety and effectiveness in real-world clinical settings.

Moreover, standardized protocols are essential to address challenges such as off-target effects, reproducibility, and scalability, ensuring consistent and predictable therapeutic outcomes. These advancements could pave the way for the clinical translation of engineered MSC-EVs as a targeted and innovative therapeutic tool.

### 3.4 Batch-to-batch consistency in EV production

Variations in culture conditions, cell passage numbers, and EV isolation methods can lead to inconsistencies in EV miRNA content and functionality (Nazari-Shafti et al., 2020). To ensure reproducibility in clinical applications, it is critical to address batch-to-batch variability. Advanced analytical techniques, such as next-generation sequencing and proteomics, should be employed to characterize miRNA profiles across different batches (Roura and Bayes-Genis, 2019). Additionally, refining standard operating procedures for MSC culture, EV isolation, and storage conditions will help reduce variability and ensure consistent and reliable EV production. This approach is essential for the successful clinical translation of MSC-EV-based therapies, offering effective treatments for a range of diseases.

## 4 Conclusion and prospects

**FIGURE 1:**
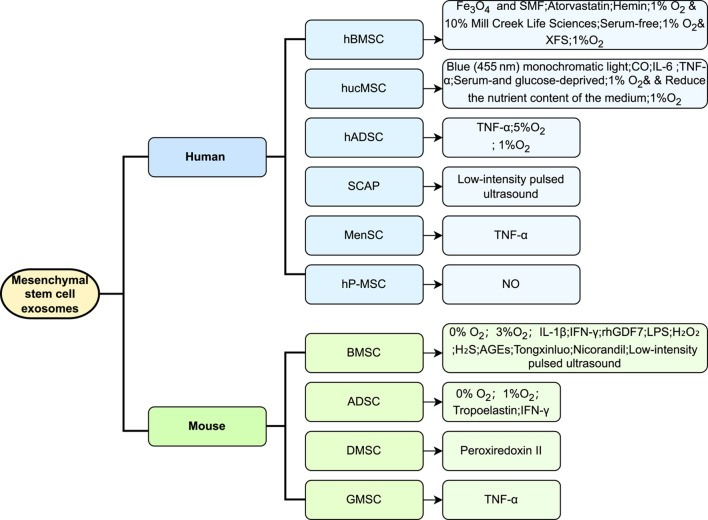
Overview of preconditioning strategies for extracellular vesicles derived from MSCs of different origins. The figure summarizes current preconditioning approaches for MSCs from various sources, such as bone marrow, adipose tissue, and umbilical cord. These strategies include hypoxia, pharmacological treatments, chemical stimulation, and physical stress, aiming to enhance the biological properties and therapeutic efficacy of EVs they produce. Differences in response to preconditioning between MSC sources are highlighted, emphasizing the importance of source-specific optimization in future applications.

MSC-EV therapy is distinguished by its stability and its ability to address various safety concerns including oncogenicity, immunogenicity, and genomic variability. This therapeutic approach has demonstrated significant potential (Racchetti and Meldolesi, 2021) in managing a broad spectrum of inflammatory (dos Santos et al., 2024) and immune (Liu et al., 2021) disorders. The efficacy of each preconditioning method varies, influenced by the treatment objectives, desired outcomes, and the origin of the MSCs. Under diverse stimulation conditions, the exosomes produced by MSCs show an enhanced expression of multiple miRNAs, which are consistently validated across studies for their roles in various diseases. For instance, the miR-21 family is recognized for its implications in various cancers, influencing lipid metabolism (Baer et al., 2013), autophagy (Yang et al., 2015b), and apoptosis (Sadri Nahand et al., 2021). The miR-146 family, which includes miR-146a and miR-146b, modulates immune and inflammatory responses (Paterson and Kriegel, 2017) and exhibits cancer-suppressive effects in pathways such as those involving platelet-derived growth factor and NF-κB (Liu et al., 2015). The miR-125 family, crucial for immune defense and hematopoietic regulation (Sun et al., 2013), and miR-126, significant for vascular endothelial cell function and inflammation reduction (Liao et al., 2024), are also of interest (Shaham et al., 2012). The regulatory roles of miR-126 and the miR-17/92 family in endothelial cells, alongside the impact of the miR-143/145 family and miR-21 in smooth muscle cell regulation, are particularly relevant in regenerative medicine (Liao et al., 2024). The pre-conditioning approaches mentioned in the paper and the corresponding changes in miRNAs, especially their targets and functions, are summarized (Table 1). It is noteworthy that the extensive validation of the role and mechanism of a specific miRNA may lead to future study focus that inadvertently neglects the exploration of other miRNAs. Accordingly, we should pursue a balanced approach to miRNA study, ensuring that while certain miRNAs are rigorously characterized, the exploration of other miRNAs is not neglected, thus preserving a wide-ranging focus in the field. To provide an intuitive overview, we present a flow chart summarizing the current state of research on different pretreatment methods applied to MSCs and their effects (Figure 1).

The expression of miRNAs in exosomes exhibits variability not only at the level of individual miRNAs but extends to broader patterns, although studies have often concentrated on miRNAs associated with specific diseases. This indicates that a more holistic approach is warranted in future investigations, especially in determining the potential role of MSC-derived EVs, produced under external stimuli, in cancer therapy. Immune checkpoint blockade (ICB) is pivotal in the tumor immune microenvironment, and the exploration of miRNA targeting to augment ICB efficacy in cancer treatment is a promising area of research (Zhang et al., 2024b). Gene-editing technologies such as CRISPR/Cas9 offer promising avenues for the precise regulation of miRNA expression in MSC-EVs, thereby enhancing their therapeutic potential. Moreover, the integration of these advanced techniques with traditional preconditioning methods could optimize the therapeutic efficacy of MSC-EVs, particularly in cancer therapy where ICB is critical. In conclusion, MSC-EV therapy is advancing toward clinical application as a promising cell-free therapeutic modality (Zhao et al., 2021). However, the lack of standardized protocols for MSC-EV preconditioning and purification remains a significant challenge. Future research should focus on elucidating miRNA changes under diverse preconditioning strategies to develop more targeted therapeutic approaches and improve EV purification techniques. Expanding MSC-EV applications to a broader range of diseases, including cancer, immune disorders, and degenerative conditions, is also crucial. Integrating advanced technologies, such as gene editing and innovative purification methods like microbead-based immunocapture, can enhance therapeutic efficacy and facilitate clinical translation. These advancements will help bridge the gap between experimental research and clinical application, unlocking the full potential of MSC-EVs for personalized and effective therapies.
